# How to Admomish

**Published:** 1878-08

**Authors:** 


**How to Admonish.**—We must consult the gentlest manner and softest seasons; for advice must not fall like a violent storm, bearing down and making those to droop whom it is meant to cherish and refresh. It must descend as dew upon the tender herb, or like melting flakes of snow; the softer it falls, the longer it dwells upon and deeper it sinks into the mind. If there are few who have the humility to receive advice as they ought, it is often because there are as few who have the discretion to convey it in a proper vehicle, and to qualify the harshness and bitterness of reproof, against which corrupt nature is apt to revolt, by an artful mixture of sweet and pleasant ingredients. To probe the wound to the bottom, with all the boldness and resolution of a good spiritual surgeon, and yet with all the delicacy and tenderness of a friend, requires a very dexterous and masterly hand. An affable deportment and a complacency of behavior will disarm the most obstinate. Whereas, if, instead of pointing out their mistake, we break out into unseemly sallies of passion, we cease to have any influence over them, or rather create a feeling antagonistic to the advice we wish to give them.

